# Supplementary material for: Complexome profiling of the Chlamydomonas psb28 mutant reveals TEF5 as an early PSII assembly factor
Source: Plant Cell. 2025 Mar 17;37(6):koaf055. doi: 10.1093/plcell/koaf055 (PMC12164590; doi:10.1093/plcell/koaf055)
Supplement: koaf055_Supplementary_Data [file koaf055_supplementary_data.zip › psb28_interactive.zip!/plots/intensity/qualityPlots/peptides/_Cre16.g659900.t1.1.html]

### Cre16.g659900.t1.1

Trivial name:   
SeqCoverage [median]: 43.60  
Mass: 24.96 kDa  
  
 

| Sequence | MissedCleavage | Mass [Da] | wtIntensity | muIntensity | Score |
| --- | --- | --- | --- | --- | --- |
| DFDLSR | 0 | 751.35 | 0.00 | 4697.88 | 111.28 |
| FVVKPPVGVAK | 1 | 1139.71 | 2995.36 | 3875.79 | 77.66 |
| ITSSLLDNLPITEAEKR | 1 | 1899.03 | 0.00 | 10740.39 | 121.32 |
| RGGGMDDETDRPNYAIALESLQNMEPEQIDR | 2 | 3519.60 | 0.00 | 1546.75 | 73.94 |
| TVMMELQGLDFPR | 0 | 1535.75 | 0.00 | 2431.93 | 125.54 |
| VVVDQLMTGQEADINDVEGLK | 0 | 2272.13 | 0.00 | 3723.76 | 147.17 |
|  |  |  | 2995.4 | 27016.5 |  |
